# Mapping HPV 16 Sub-Lineages in Anal Cancer and Implications for Disease Outcomes

**DOI:** 10.3390/diagnostics12123222

**Published:** 2022-12-19

**Authors:** Daniel Guerendiain, Laila Sara Arroyo Mühr, Raluca Grigorescu, Matthew T. G. Holden, Kate Cuschieri

**Affiliations:** 1 Scottish HPV Reference Laboratory, Royal Infirmary of Edinburgh, 51 Little France Crescent, Edinburgh EH16 4SA, UK; 2 School of Medicine, University of St Andrews, St Andrews KY16 9TF, UK; 3 International HPV Reference Center, Department of Laboratory Medicine, Karolinska Institutet, 141 86 Stockholm, Sweden; 4 Department of Pathology, Royal Infirmary of Edinburgh, 51 Little France Crescent, Edinburgh EH16 4SA, UK

**Keywords:** human papillomavirus, HPV 16 sub-lineages, anal cancer

## Abstract

The incidence of anal cancer is rising worldwide. As identified in cervical cancer management, an improvement in the early detection and management of anal pre-cancer is essential. In other cancers associated with human papillomavirus (HPV), HPV 16 sub-lineages have been shown to be associated with disease status and prognosis. However, in anal cancer, they have been under-explored. A total of 119 HPV 16-positive anal cancer lesions diagnosed between 2009 and 2018 in Scotland and 134 HPV 16-positive residual rectal swabs from asymptomatic men collected in 2016/7 were whole genome sequenced. The association of HPV 16 sub-lineages with underlying disease status (cancer vs. asymptomatic) and overall survival in anal cancer samples was assessed (comparing A1 vs non-A1 sub-lineages). A1 was the dominant sub-lineage present in the anal cancer (76.5%) and the asymptomatic (76.1%) cohorts. A2 was the second most dominant sub-lineage in both groups (16.8% and 17.2%, respectively). We did not observe significant associations of sub-lineage with demographics, clinical variables or survival (A1 vs. non-A1 sub-lineages (HR 0.83, 0.28–2.46 *p* = 0.743)). HPV 16 sub-lineages do to not appear to cluster with disease vs asymptomatic carriage or be independently associated with outcomes in anal cancer patients. Further international studies on anal HPV sub-lineage mapping will help to determine whether this is a consistent observation.

## 1. Introduction

Anal cancer is one of the six cancers shown to have a human papillomavirus (HPV) aetiology [1]. Most HPV-positive anal cancers are caused by HPV type 16 (HPV 16), and in a recent population-based assessment in Scotland, in cases diagnosed between 2009–2018, HPV 16 was detected in 93.3% of the HPV positive cases [2], higher than the amount of HPV 16 attributable to cervical cancer [3].

Additionally, as with other HPV-driven cancers, anal cancer incidence is increasing worldwide, including in the USA and Europe [4,5,6,7].

HPVs are formally classified as “types” based on the nucleotide sequence of the open reading frame (ORF) coding for the major capsid protein: L1 [8]. HPV types differ by more than 10% of their primary sequence compared to their most closely related type [8]. Phenotypic differences in HPV types with respect to disease risk and tissue tropism are well-established, and this knowledge has informed the development of effective vaccines and HPV-based cervical screening assays. However, below the level of HPV type exist lineages (with 2–10% variation) and sub-lineages (0.5% to 2% variation) [9], and the implications of this level of variation on clinical outcomes of infection is less established.

For HPV 16, four lineages have been identified (lineages A, B, C and D), as well as 16 sub-lineages: A, including A1–A3 (previously named European) and A4 (Asian) sub-lineages; B, including B1 (African-1, Afr1a) and B2 (African-1, Afr1b), B3 and B4 sub-lineages; C1 (African-2, Afr2a), C2, C3 and C4; and D, including D1 (North American, NA1), D2 (Asian-American, AA2), D3 (Asian-American, AA1) and D4 sub-lineages [9].

Although some investigators have assessed the global distribution of sub-lineages, the majority have focused on cervical cancers rather than other HPV-driven cancers. In 2013, Cornet et al. looked at the HPV lineages in cervical cancers and showed that European sub-lineages (A1–A3) were the most common in all regions of the world, except in sub-Saharan Africa and East Asia, whereas the African sub-lineages dominated in the northern sub-Saharan region of Africa, and the Asian variant in East Asia [10]. Nicolás-Párraga et al. (2016) found similar results, with A1–3 present in 95.65% of the cases in Europe, 78.26% in Central/South America (D in 21.73%) and 80% in Asia (12% A4 and 7.69% D) [11].

In terms of HPV 16 sub-lineages present in the anus, data is relatively sparce. Volpini et al. (2017) investigated the HPV 16 variants in anal samples collated in Brazil, finding that 70.8% were classified as A1–3 sub-lineages and 29.2% as “other” [12]. A recent systematic review, performed by Ferreira et al. (2021) of genetic variants of HPV-16 in men, found HPV 16 lineages vary according to anatomical and geographical regions, but they found that European samples had a high prevalence (86.59%) of HPV 16 lineage A [13].

In the context of cervical disease, evidence suggests that sub-lineages and variants may be independently associated with poor clinical outcomes. Mirabello et al. (2015) [14] revealed a higher risk of disease associated with B/C/D lineages as a group compared to the A lineage. Clifford et al. (2019). also found an increased cervical cancer risk for A3, A4 and D-(sub-) lineages vs the A1 sub-lineage. A more recent study by Lang Kuhs et al. (2022) looked into the genetic variation of HPV 16 and its association with clinical outcomes in HPV 16-positive oropharyngeal cancer patients. They investigated different high-risk single nucleotide polymorphisms (SNPs) and found that those with one or more high-risk SNPs had a median survival time of 3.96 years compared to 18.67 years for those with no high-risk SNPs. Most of these SNPs were common to the D2 sub-lineage, which have also been associated with higher risk of cancer in the cervix [14]. However equivalent studies on anal cancer are rare.

We recently identified that the viral load of HPV16 in anal cancer may be informative for prognosis [2]. Now, due to the information published on the association of HPV 16 sub-lineages and cancer risk yet the comparative absence of data in the anal context, we aimed to better understand the pattern and dominance of HPV 16 sub-lineages in a population-based cohort of anal cancer and to determine whether significant associations with sub-lineage and demographic or clinical variables existed). Data obtained from the cancer cohort was contextualized and compared to variant profile in anal samples obtained from an asymptomatic population.

## 2. Material and Methods

### 2.1. Sample Collection

A total of 150 HPV 16-positive anal cancers and 182 DNA extracts from residual rectal swabs obtained from asymptomatic men were selected for HPV 16 sub-lineage identification through whole genome sequencing (WGS).

#### 2.1.1. Anal Cancer Cohort, Collection and Annotation

For the present work, we used the same anal cancer (*n* = 150) sample set as described in detail in Guerendiain et al. (2022) [2]. Briefly, nucleic acid extract associated with archived formalin-fixed, paraffin-embedded tissue was genotyped using the Seegene Anyplex II 28 (Seoul, Korea), followed by storage at −80 °C. Anal cancer biopsy samples were taken between 2009 and 2018 as part of the management of patients with anal disease from 3 of the 14 territorial health boards in Scotland (NHS Lothian, NHS Borders and NHS Fife).

HPV typing was performed at the Scottish HPV Reference Laboratory, Edinburgh, UK. One 10 μm section per sample was obtained and incubated in Seegene Universal Lysis Buffer (LB) at 65 °C overnight. DNA extraction was performed using the Microlab Nimbus IVD (Hamilton, Reno, USA) with the StarMAg Universal cartridge Kit (Seegene), following manufacturers’ instructions. Mastermix was prepared with the Nimbus and PCR on the CFX Real-Time PCR instrument (Biorad, CA, USA).

As described in Guerendiain et al. (2022), clinico-demographic information was obtained in January 2020, specifically the patient’s age, sex, stage of cancer (using the American Joint Committee on Cancer (AJCC) TNM system) [15], response to treatment, date of diagnosis and vital (dead/alive) status. Age and stage of cancer were considered at the time of diagnosis. Vital status information and date of death data was censored in July 2020.

Cases categorized according to the various clinical and demographic variables are summarized in Table 1. Age was stratified in 4 different groups: <50, 50–59, 60–69 and ≥0. Response to treatment was organized in 3 groups: yes, no or unknown, following the ESMO guidelines for anal cancer [16]. Cancer stage was aggregated in 5 groups: I, II, III, IV and unknown, following the AJCC system effective January 2018 [15].

#### 2.1.2. Residual Rectal Swabs from Asymptomatic Men

To contextualize the sequences observed in the anal cancers, a disease-free control group of anonymized residual rectal swabs obtained from asymptomatic men attending sexual health clinics were collated for downstream WGS. These samples had previously been genotyped as a consequence of immunization surveillance in Scotland [17]. DNA was extracted from the residual rectal swabs by Qiagen MDx (Hilden, Germany) or Seegene Universal Extraction System, obtaining an eluate volume of 100 μL. HPV genotyping was performed using the Seegene Anyplex II and the Optiplex HPV Genotyping Kit (Heildelberg, Germany), detecting 28 and 24 different HPV types, respectively.

A total of 182 anonymized DNA extracts from residual rectal swabs were collated. Samples had originally been collected in the years 2016–2017 and were included if positive for HPV 16.

### 2.2. Governance

Use of samples for the present project was approved by the Southeast of Scotland National Research for Scotland Bioresource (NRS) (application reference SR 1283 and SR1364). A favorable ethical opinion to conduct the research was provided by University of St Andrews Teaching and Research Ethics Committee, reference MD 14482.

### 2.3. PCR Target-Enrichment for Deep Sequencing of HPV 16

HPV 16 whole genome material was amplified using 47 overlapping amplicons described in Cullen et al. [18] and optimized by Arroyo et al. [19] Briefly, primer sets were divided into five different reactions to decrease self-dimer and cross-primer dimer formation. PCRs were performed using Qiagen Multiplex PCR Master Mix (Qiagen, Hilden, Germany) and 0.2 µM of each primer, according to manufacturers’ instructions. PCR amplification products were pooled together according to sample name prior to library preparation.

### 2.4. Library Preparation

Libraries were prepared using the Illumina DNA prep kit (San Diego, CA, USA) following the manufacturer’s instructions, using 450 ng of DNA in 35 μL as input. Sequencing was performed using the Illumina MiSeq instrument and the Illumina MiSeq reagent kit v2 500 cycles (2 × 250 bp). Libraries were normalized to 4 nM in combination with 12.5 pM of PhiX (Illumina).

### 2.5. Quality Control and Quality Analysis

HPV 16-positive (SiHa) and HPV-negative (water) controls were added at the DNA extraction step and carried through the PCR, library preparation and data analysis stages. Individual amplification products were assessed using a Bioanalyzer (Santa Clara, CA, USA). Quality control for library preparation included both controlling the library size using an Agilent Tapestation (Santa Clara, CA, USA) and determining the DNA concentration using the Qubit dsDNA High-Sensitivity Assay Kit (ThermoFisher, Waltham, MA, USA).

As a further quality control for analysis of the sequence data generated, a subset of 25 fastq files were sent to the International HPV Reference Laboratory in Karolinska, Sweden for independent bioinformatic analysis and sub-lineage identification.

### 2.6. Bioinformatic Analysis

Reads obtained from Illumina were de-multiplexed and converted to fastq files. All fastq files were quality and adaptor trimmed using Trimmomatic (v0.39) [20]. Only high-quality paired reads (-phred 33 -leading 3 -trailing 3- slidingWindow: 4:15) with 150 bp were used for further analysis. FASTQC tools were further used to assess whether any adaptors remained [21]. High-quality reads were then mapped to the HPV 16 reference genome from the Papillomavirus Episteme (PaVE) [22] using bwa (v0.7.17) [23], to create a sam file. Due to the circular HPV genome, the reference genome was modified by adding the 258 nucleotides from the beginning to the end of the genome sequence to not lose coverage of amplicons 46 and 47. SAMtools (v1.14) [24] was then used to convert files from sam to bam and to curate files for the variant calling. BCFtools (v1.14), mpileup and consensus tools were used for the variant calling and for the generation of a consensus sequence [25], using default parameters. Positions not covered were annotated as Ns.

New consensus files were aligned using MAFFT (v7.490) with default parameters [26]. A manual edit was performed when required. Maximum likelihood trees were inferred using RaxML (v2.0.8) [27] with the GTR substitution model (ML + transfer bootstrap expectation + consensus, 1 run, 100 reps). Visualization of the trees generated by RaxML was performed using Figtree (v1.4.4). Each sample was assigned with a sub-lineage corresponding to the nearest neighbor.

Sub-lineages references were obtained from the PAVE for each of the HPV 16 sub-lineages: A1 (K02718.1), A2 (AF536179.1), A3 (HQ644236.1), A4 (AF534061.1), B1 (AF536180.1), B2 (HQ644298.1), B3 (HQ644298.1), B4 (KU053914.1), C1 (AF472509.1), C2 (HQ644244.1), C3 (KU053920.1), C4 (KU053925.1), D1 (HQ644257.1), D2 (AY686579.1), D3 (AF402678.1) and D4 (AF402678.1) A sub-lineage assignment was performed for all specimens excluding those with <100× median depth or low genome coverage (<80% genome coverage).

### 2.7. Assessment of Variants According to Clinic-Demographic Characteristics and Survival Analysis

To assess the relationship between HPV sub-lineages and different factors (two or more independent variables), a univariate logistic regression analysis was performed between HPV 16 sub-lineages (HPV 16 A1-positive vs. HPV 16 non-A1-positive), age at diagnosis, collection year and health board of diagnosis. Adjustment was performed for age group (<50, 50–59, 60–69 and 70 or over), sex, response to treatment, stage of cancer and vital status (dead or alive). Comparison was performed between A1 vs. non-A1 sub-lineages due to the small number of samples identified from the different sub-lineages. The non-A1 group includes the following sub-lineages: A2, A3, A4, B1, B2, B3, B4, C1, C2, C3, C4, D1, D2, D3 and D4.

Odds ratios (OR) were calculated to quantify the strength of the association between HPV 16 sub-lineages and the demographic and clinical data. All the statistics were obtained using R-studio macOS, (version 1.2.1335) [28]. The distribution of sub-lineages in anal cancers vs. the asymptomatic population was assessed with sequences from the two groups displayed in a phylogenetic tree.

Overall survival by HPV 16 sub-lineages (HPV 16 A1-positive vs. HPV 16 non-A1-positive) was analyzed using the Kaplan-Meier method. The univariate and multivariate hazard ratios of HPV 16 sub-lineages (HPV 16 A1-positive vs. HPV 16 non-A1-positive) for all-cause death were derived using the cox proportional hazard model. A univariate and multivariate model was derived; age (<50, 50–59, 60–69, 70+), sex, stage (I, II, III, IV) and response to treatment (no, yes) were adjusted for. All the statistical analyses were performed using R-studio (version 1.2.1335) [29]. Differences in prevalence of the HPV 16 sub-lineages between the anal cancer cohort and asymptomatic control cohort are presented descriptively.

## 3. Results

A total of 182 asymptomatic/control samples and 150 anal cancer samples were subjected to WGS. In the anal cancer cohort, 119/150 (79.3%) samples passed the quality parameters, and in the asymptomatic men cohort, 134/182 (73.6%) were valid. This left a total of 253 samples for inclusion for detailed sequencing/phylogenetic analysis. Twenty-five of these sequences were also analyzed by the International HPV Reference Center as a further quality control for analysis. Results showed 100% agreement.

### 3.1. Distribution of HPV 16 Sub-Lineages in Anal Cancers

**Figure 1:**
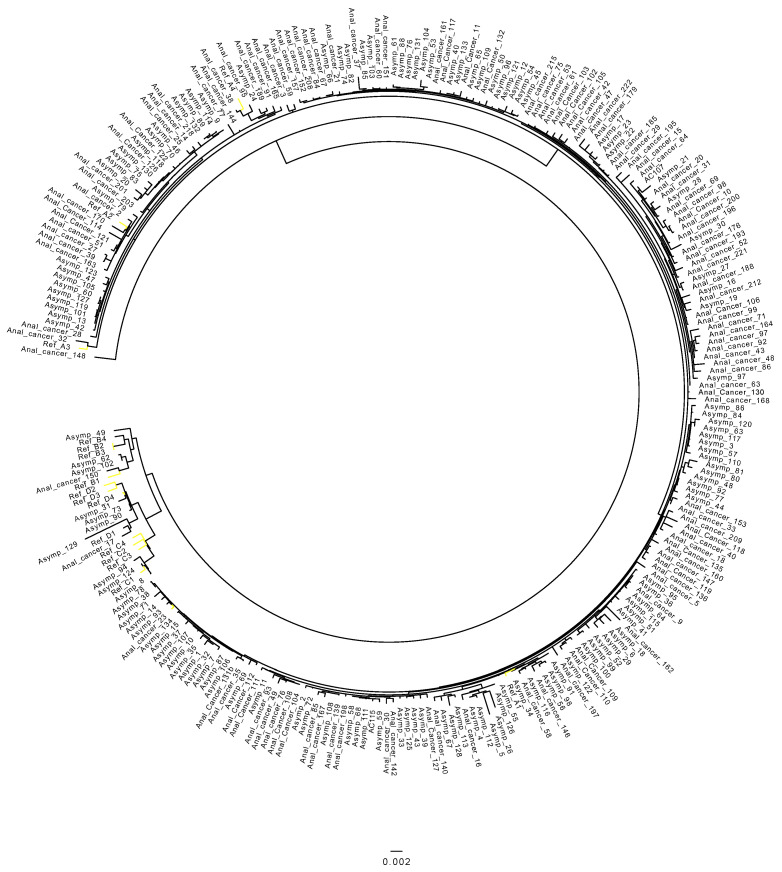
Phylogenetic tree representing the HPV 16 sub-lineages present in the anal sample and control groups.

Of the 119 cancer cases with sufficient read depth (>100× median depth and >80% genome), the HPV 16 sub-lineage A1 was identified in 91 anal cancer samples (76.5%), followed by A2, which was identified in 20/119 (16.8%) of samples. A4 was detected in 5/119 samples (4.2%). Two samples were classified as B1 (1.7%), one as A3 (0.8%) and one as D1 (0.8%). Further detail of HPV 16 sub-lineages in anal cancers is described in detail in Table 2 and Figure 1.

### 3.2. HPV 16 Sub-Lineages in the Control Cohort

Of the 134 control samples, most samples were classified (76.1%) as A1, followed by A2, identified in 23/134 (17.2%) of samples. D1 sub-lineage was identified in 4/134 samples (3.0%), and C1 and B1 were identified in two cases each (1.5%). B2 was present in 1/134 (0.7%). Table 2 describes the number of cases identified for each sub-lineage, and Figure 1 contains the phylogenetic tree obtained from the control cohort.

### 3.3. Differences in Prevalence of HPV 16 Sub-Lineages between Anal Cancer and Control Cohort

No major differences in the proportion of A1 and A2 sub-lineages were observed between the case vs. control cohorts, being 76.4% vs. 76.0% and 16.2% vs. 17.0%, respectively. Whereas the case cohort revealed the presence of the A4 sub-lineage in 4.2% of anal cancers, this sub-lineage was not present in the control cohort. Conversely, the C lineage was present in 1.5% of control specimens and was not detected in cancer cases. Finally, there was a slight increase in the D1 sub-lineage observed in the control vs. case cohort (3.0% vs. 0.8%).

### 3.4. Association of HPV 16 Sub-Lineages with Demographic and Clinical Variables

From the 119 anal cancers, four samples did not contain vital status information and were not included in the analysis. Due to the dominance of the A1 sub-lineage, the logistic analysis and odds ratio analysis were performed based on the presence or absence of the HPV 16 sub-lineage A1.

No significant differences in A1 positivity with sex, age, response to treatment, stage or vital status were observed. This observation was consistent for the adjusted analysis (Table 3).

### 3.5. HPV 16 Sub-Lineages and Overall Survival

**Figure 2:**
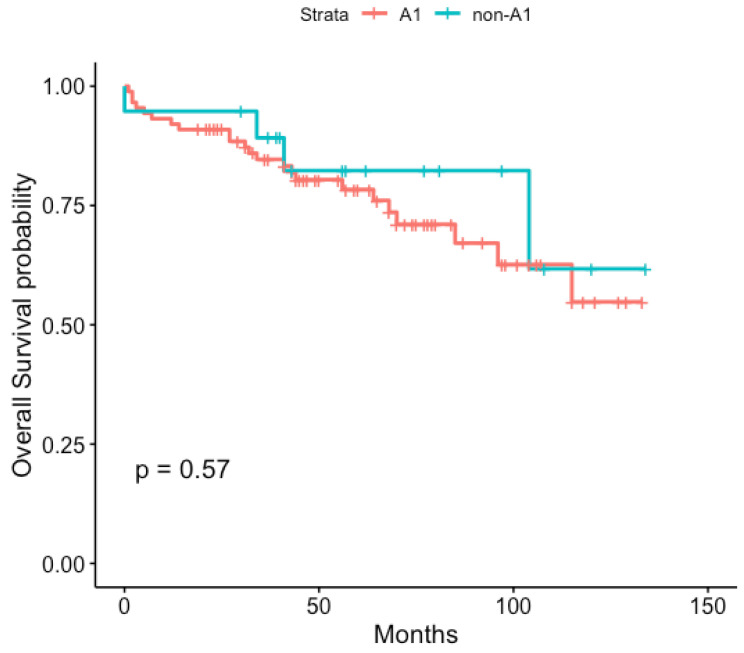
Kaplan-Meier survival curve stratified by HPV 16 sub-lineages (A1 vs. Non-A1). Survival time expressed in months from the diagnosis date. Data censored on 31 July 2020.

For the Kaplan-Meier estimator, overall survival was calculated by classifying HPV 16 sub-lineages into A1 presence or absence. No differences in overall survival were found between both sub-lineage groups (*p* = 0.57), Figure 2.

Table 4 shows overall survival stratified by the clinical and demographic variables (age group, sex, cancer stage and response to treatment), with HPV 16 sub-lineages categorized into the two groups (A1 vs. non-A1) with A1 as the reference. Non-A1 (vs. A1) was not associated with improved overall survival in the univariate analysis, with a hazard ratio (HR) of 0.87 (0.37–2, *p* = 0.751). Variables associated with worse overall survival in the univariate model were stage IV vs. stage I with HR of 15.7 (3.38–72.8), *p* < 0.001 and response to treatment vs. no response to treatment with HR of 0.11 (0.05–0.25) *p* < 0.001. After adjustment for age, gender, stage and response to treatment, non-A1 sub-lineages did not significantly influence the overall survival compared to A1, with a HR 0.83 (0.28–2.46, *p* = 0.743).

If only A1 and A2 samples were considered, no significant differences in HR were found when using A1 as the reference (HR 0.74, 0.25–2.1, *p* = 0.575).

### 3.6. Integration

Although it was not the main aim of the study, the absence of part of the HPV 16 genome was identified in 13/119 (10.92%) of the anal cancer samples. This absence indicates the potential integration of the HPV16 in the human genome. The E2 gene was the most frequently missing region, followed by E4, E5 and L2 E1 and L1 (see Table 5 for details). Notably, all cases retained E6 and E7 oncogenes. Due to the small number of cases in which integration was detected, no further analysis was performed. No integration was detected in the asymptomatic cohort.

## 4. Discussion

Previously, we described that 93.3% of HPV-positive anal cancer cases diagnosed in Scotland between 2019 and 2018 were caused by HPV 16. In this study, we have identified that 76% of cases belonged to the A1 sub-lineage, followed by A2 (16%).

In the control group of asymptomatic men, a similar prevalence of A1 and A2 was observed. Differences identified were the presence of A4 in the anal cancers (4.7%), which was absent in the control group; presence of the C lineage only detected in the control group; and the presence of the sub-lineage D1 in the control group (3%), which had a lower prevalence of 0.81% in the cancers. This higher prevalence of sub-lineages A1 and A2 is consistent with previously published studies in European cohorts. Gonçalves et al. (2022) found a higher prevalence of the A lineage in the anal canal of asymptomatic men, mainly A1 [29], and Nicolás-Párraga et al. (2016) found that A1–3 sub-lineages were identified in 96.1% of the European cases [30]. Beyond Europe, Volpini et al. (2017) investigated the HPV 16 variants in cervical and anal samples collated in Brazil and determined that proportionally less of the anal cancer samples (70.8%) were classified as A1–3 sub-lineages [12].

The data collated in the study add to the limited information on the pattern and implications of HPV sub-lineages in the anus. Though we did not see significant associations with demographic and underlying disease status, these observations need to be confirmed or refuted by future studies with larger sample sizes.

To our knowledge, no other studies have investigated the association of HPV 16 sub-lineages in anal cancer and overall survival. We did not observe that A1 vs. non -A1 sub-lineages influenced overall survival in the univariate and adjusted analysis. Interestingly, a recent study was reported by Lang Kuhs et al. (2022) in which the authors looked into the genetic variation of HPV 16 and its association with clinical outcomes in HPV 16-positive oropharyngeal cancer patients [31]. They investigated different high-risk single nucleotide polymorphisms (SNPs) and found that those with one or more high-risk SNPs had significantly shorter median survival times. Most of these SNPs were common to the D2 sub-lineage, which has also been associated with a higher risk of cancer in the cervix [14]. Due to the absence of D2 cases in the present study, we were not able to explore this in the present work; however, the identification of these high-risk SNPs may be very helpful for patient and treatment management.

Although we identified potential integration of the HPV16 genome (calculated through the loss of the sequence), due to the small number, we did not perform any further analysis, including in relation to implications for survival. Given the relative lack of information on the extent and implications of integration in anal cancer, we would assert that this is an area that would benefit from further study.

We acknowledge this study has limitations; the asymptomatic population were all men, whereas the cancer population had a majority of female (75.63%) samples compared to males (24.37%); this was due to pragmatic reasons relating to available material. However, data did not show differences in the distribution of HPV 16 sub-lineages between women and men in the anal cancer group. Additionally, as discussed earlier, we believe the observations made in the present work would benefit from validation in a larger sample of cases and controls and would hope this study serves as a primer for such. Though the number of cases of cancers was not trivial (*n* = 253), particularly given that the Scottish European age-standardized rate (EASR) (per 100,000 person-years at risk) was 2.6 in 2017, we appreciate that detecting rarer sub-lineages with precision can take large sample sizes.

In the UK, there is no screening program for anal cancer. However, since 2017, there has been an opportunistic vaccination program for MSM, and in 2019, the national HPV vaccination became gender neutral. In term of vaccines, a study from Godi et al. (2019) reported that HPV 16 lineage variants B, C and D exhibited slightly (<two-fold) reduced sensitivity to nonavalent vaccine sera compared to lineage A [32].

Therefore, the high prevalence of lineage A in the samples included in this study could be interpreted as positive for vaccine efficacy, particularly given that gender-neutral vaccination is now a part of core policy in the UK and several other countries.

This study has demonstrated the technical feasibility of detecting HPV 16 sub-lineages in anal cancer samples and residual material from rectal swabs. Though some differences in the presence of non-A sub-lineages were detectable between the cancer and asymptomatic population, the consistency, magnitude and implications of these would benefit from further study. The domination of lineage A is consistent with existing European data and suggests that sub-lineage identification in itself may not be informative for prognostication.

## Figures and Tables

**Table 1:** Anal cancer cohort: clinical characteristics and demographics with valid NGS analysis.

Variable	Level	*n* = 119	%
Sex	Female	90	75.6
	Male	29	24.4
Age	<50	15	12.6
	50–59	32	26.9
	60–69	39	32.8
	70 and over	33	27.7
Stage	I	18	15.1
	II	48	40.3
	III	36	30.2
	IV	16	13.4
	Unknown	1	0.8
Response to treatment	Yes	95	79.8
	No	17	14.3
	Unknown	7	5.9
Vital status	Alive	87	73.1
	Deceased	30	25.2
	Unknown	2	1.7

**Table 2:** HPV 16 sub-lineages identified in the anal cancer and asymptomatic cohorts.

	Anal Cancer		Asymptomatic Group	
Sub-Lineage	*N*	% (*N* = 119)	*N*	% (*N* = 134)
HPV 16 A1	91	76.5%	102	76.1%
HPV 16 A2	20	16.8%	23	17.2%
HPV 16 A3	1	0.8%	0	0%
HPV 16 A4	5	4.2%	0	0%
HPV 16 B1	2	1.7%	2	1.5%
HPV 16 B2	0	0.0%	1	0.7%
HPV 16 B3	0	0.0%	0	0%
HPV 16 B4	0	0.0%	0	0%
HPV 16 C1	0	0.0%	2	1.5%
HPV 16 C2	0	0.0%	0	0%
HPV 16 C3	0	0.0%	0	0%
HPV 16 C4	0	0.0%	0	0%
HPV 16 D1	1	0.8%	4	3.0%
HPV 16 D2	0	0.0%	0	0%
HPV 16 D3	0	0.0%	0	0%
HPV 16 D4	0	0.0%	0	0%
Total	119		134	

**Table 3:** Influence of A1 sub-lineage presence stratified by demographic and clinical variables. The comparator for the odds ratio (univariate and adjusted) is A1 absence.

Variable	Level	Unadjusted OR (95% Cis)	*p* Value	Adjusted OR (95% Cis)	*p* Value
Sex	Male	1		1	
	Female	1.12 (0.39–2.92)	0.827	1.09 (0.37–3.00)	0.87
Age	<50	1		1	
	50–59	1.20 (0.27–4.80)	0.8	1.11 (0.24–4.56)	0.89
	60–69	1.50 (0.34–5.93)	0.57	1.82 (0.40–7.67)	0.416
	70 and over	1.37 (0.30–5.67)	0.666	1.63 (0.34–7.41)	0.529
Response to treatment	No	1		1	
	Yes	1.02 (0.26–3.26)	0.968	1.18 (0.34–7.41)	0.528
Stage	I	1		1	
	II	1.36 (0.37–4.62)	0.625	1.28 (0.33–4.51)	0.706
	III	1.60 (0.40–6.11)	0.486	1.56 (0.38–6.06)	0.522
	IV	1.80 (0.36–10.40)	0.478	3.03 (0.42–29.47)	0.289
Vital Status	Alive	1		1	
	Deceased	1.01 (0.39–2.85)	0.983	0.92 (0.25–3.81)	0.907

**Table 4:** Hazard ratio of HPV 16 sub-lineages (univariate and multivariate) derived from Cox regression (*N* = 115) in anal cancer samples collected between 2009 to 2018 in the southeast of Scotland.

Variable	Level	Unadjusted HR (95% Cis)	*p* Value	Adjusted HR (95% Cis)	*p* Value
HPV 16 sub-lineage	A1 (*n* = 88)	1		1	
	Non-A1 (*n* = 27)	0.87 (0.37–2)	0.751	0.83 (0.28–2.46)	0.743
Sex	Male	1		1	
	Female	1.2 (0.48–2.9)	0.71	0.88 (0.32–2.39)	0.795
Age	<50	1		1	
	50–59	1.10 (0.33–3.70)	0.877	0.83 (0.21–3.26)	0.788
	60–69	0.85 (0.26–2.8)	0.795	2.67 (0.607–11.72)	0.194
	70 and over	1.54 (0.48–5.0)	0.466	5.56 (1.082–28.58)	0.04
Stage	I	1		1	
	II	1.7 (0.37–8.1)	0.49	2.34 (0.47–11.74)	0.302
	III	2.4 (0.50–11.6)	0.274	2.26 (0.42–12.27)	0.344
	IV	15.7 (3.38–72.8)	<0.001	15.95 (2.45–10.3.82)	0.004
Response to treatment	No	1		1	
	Yes	0.11 (0.05–0.25)	<0.001	0.12 (0.03–0.39)	<0.001

**Table 5:** HPV integration identified in the anal cancer cohort.

HPV Genes Integration in the Anal Cancer Samples (*n* = 13)	*N*
L1 only	1
E1 only	1
E1, E2, E4	1
E2, E5, part E2	1
E2, E4, E5, L2, L1	3
E1, E2, E4, E5 and part L2	2
E1, E2, E4, E5, L2 and part L1	3
E1, E2, E4, E5, L2 and L1 complete	1

## Data Availability

Request for data in anonymized form can be made available upon reasonable request to the senior author and following due process of governance and the Scottish Data Protection Regulations. GenBank submission IDs 2637056 and 2638666.

## Abbreviations

Human papillomavirus (HPV), hazard ratio (HR), European age-standardized rate (EASR), human immunodefiency virus (HIV), deoxyribonucleic acid (DNA), overall survival (OS), National Health Service (NHS), whole genome sequencing (WGS).
